# An automated system for measuring parameters of nematode sinusoidal movement

**DOI:** 10.1186/1471-2156-6-5

**Published:** 2005-02-07

**Authors:** Christopher J Cronin, Jane E Mendel, Saleem Mukhtar, Young-Mee Kim, Robert C Stirbl, Jehoshua Bruck, Paul W Sternberg

**Affiliations:** 1 HHMI and Division of Biology, California Institute of Technology, Pasadena, CA, USA; 2 Computation and Neural Systems, California Institute of Technology, Pasadena, CA, USA; 3 Jet Propulsion Laboratory, Pasadena, CA, USA; 4 21018 Wendy Drive, Torrance, CA 90503, USA

## Abstract

**Background:**

Nematode sinusoidal movement has been used as a phenotype in many studies of *C. elegans *development, behavior and physiology. A thorough understanding of the ways in which genes control these aspects of biology depends, in part, on the accuracy of phenotypic analysis. While worms that move poorly are relatively easy to describe, description of hyperactive movement and movement modulation presents more of a challenge. An enhanced capability to analyze all the complexities of nematode movement will thus help our understanding of how genes control behavior.

**Results:**

We have developed a user-friendly system to analyze nematode movement in an automated and quantitative manner. In this system nematodes are automatically recognized and a computer-controlled microscope stage ensures that the nematode is kept within the camera field of view while video images from the camera are stored on videotape. In a second step, the images from the videotapes are processed to recognize the worm and to extract its changing position and posture over time. From this information, a variety of movement parameters are calculated. These parameters include the velocity of the worm's centroid, the velocity of the worm along its track, the extent and frequency of body bending, the amplitude and wavelength of the sinusoidal movement, and the propagation of the contraction wave along the body. The length of the worm is also determined and used to normalize the amplitude and wavelength measurements.

To demonstrate the utility of this system, we report here a comparison of movement parameters for a small set of mutants affecting the Go/Gq mediated signaling network that controls acetylcholine release at the neuromuscular junction. The system allows comparison of distinct genotypes that affect movement similarly (activation of Gq-alpha versus loss of Go-alpha function), as well as of different mutant alleles at a single locus (null and dominant negative alleles of the *goa-1 *gene, which encodes Go-alpha). We also demonstrate the use of this system for analyzing the effects of toxic agents. Concentration-response curves for the toxicants arsenite and aldicarb, both of which affect motility, were determined for wild-type and several mutant strains, identifying P-glycoprotein mutants as not significantly more sensitive to either compound, while *cat-4 *mutants are more sensitive to arsenite but not aldicarb.

**Conclusions:**

Automated analysis of nematode movement facilitates a broad spectrum of experiments. Detailed genetic analysis of multiple alleles and of distinct genes in a regulatory network is now possible. These studies will facilitate quantitative modeling of *C. elegans *movement, as well as a comparison of gene function. Concentration-response curves will allow rigorous analysis of toxic agents as well as of pharmacological agents. This type of system thus represents a powerful analytical tool that can be readily coupled with the molecular genetics of nematodes.

## Background

A major motivation for establishing *C. elegans *as an experimental molecular genetic system was to understand how genes control behavior, especially locomotion, since the uncoordinated (Unc) mutants were discovered early in the history of *C. elegans *genetics [1,2]. While studies of hundreds of genes involved in this behavior have led to many insights into processes such as axonal guidance (*unc-5*, *unc-6 *and *unc-40*; [3,4]), synaptic transmission (*unc-13*, *unc-18*; [5-7]), myosin assembly (*unc-54 *[8]), regulation of G protein signaling [9-16]), neuropeptide function [17] among many others, there has been no general understanding of how *C. elegans *moves. Starting with Brenner [1]*C. elegans *researchers have identified several hundred genes with effects on movement. Recently, RNAi screens have identified 1371 of 27,574 experiments (approximately 800 genes) that confer abnormal movement [18]. Normal nematode movement is indicative of a toxicant-free environment, and of youthful vigorous worms. Drugs and toxins affect worm movement [19-21], and locomotory defects are a hallmark of aging worms [22]. Models for *C. elegans *movement (e.g. [23-25]) would be enhanced by additional quantitative data.

Descriptions of movement phenotypes, particularly hyperactive locomotion, have been partial and to some extent anecdotal. For example, mutations in a number of genes result in activation of the EGL-30 (Gαq) signaling pathway and cause an increase in the frequency of body bends [7,9,10,12,15,17,26-30]. Overexpression of or gain-of-function mutations in *egl-30 *also cause animals to move with exaggerated body bends [28,29], but the presence or absence of this phenotype has only been reported for a few of the Gαq pathway activators [15,17,26]. The rate of locomotion has often been determined by manually counting body bends per minute (e.g., [30-32]). Amplitude of body waves has also been determined manually [17,26]. Keating et al. [33] used visual inspection or manual quantification to screen for movement defects caused by RNAi depletion of neuropeptide receptors. These approaches, while useful, are labor intensive and provide only a partial description of the movement of a particular genotype.

We therefore developed a system to analyze the body posture of *C. elegans *hermaphrodites over time and extract quantitative information concerning their movement. Here we describe a functional system, metrics, analysis tools, and example applications for distinguishing closely related *C. elegans *mutants and establishing concentration-response relationships for toxic agents.

There have been other developments of automated systems. For example, Williams and Dusenbery [34] tracked the centroids of multiple worms simultaneously. Several other studies have also used automatic tracking of centroids to analyze velocity, dispersal and turning rate [35-41]. Hirose et al., [42] have recently described a system to automatically measure body length, using image processing similar to that described here. At the time we developed the prototype of the system described here (1999–2000), there were no systems available. Here we describe a set of metrics that allow intuitive use of an automated system, and show the utility of these metrics for genetic studies and studies of toxic agents. During preparation of this paper, we have implemented a combined system that uses the metrics and some algorithms described here with components of a related system developed by Schafer and colleagues [43,44]. The hardware and software for the hybrid system is described by [45]; the metrics and applications are described here in relation to our system.

## Results

### The movement analysis system

Our system analyzes motion in two phases. First, video and worm posture data are acquired using the system hardware. Second, measures of behavior are extracted by software. Data acquisition is a two-step process: videotaping and data extraction.

### Videotaping

**Figure 1:**
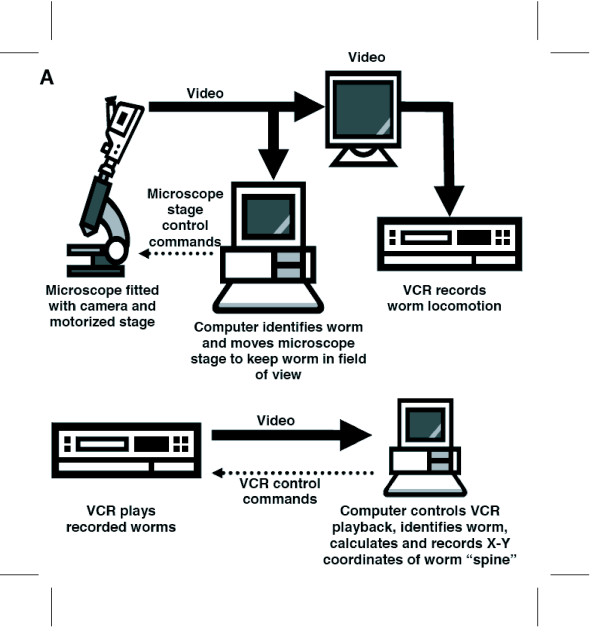
**Tracker and Recognizer Schematic**. A. Tracker. A Petri plate with worm is placed on a computer-controlled motorized stage. A joystick is used to center the worm in the field of view. The worm is recorded on VCR. B. Recognizer. The video tape is played into a computer by a computer-controlled VCR to recognize the worm and record its body posture and position as a function of time.

We assembled a videotaping apparatus comprising a personal computer with the Tracker software package that we developed, a Matrox Meteor-II/Standard video frame grabber, a Wild M5A stereo dissecting microscope with camera mount, a Dage-MTI CCD72 video camera and controller, a Sony video monitor, a Ludl Electronic Products BioPoint motorized inverted-microscope stage and controller with a joystick for manually moving the stage, a stage-mounted custom Petri dish carrier, and a Panasonic model AG-5710P VHS video cassette recorder (which has an RS-232 connection for computer control and feedback of VCR operation) (Figure 1A).

The behavior of individual worms is examined on Petri plates with fresh, uniform bacterial lawns (see Methods), conditions that favor continued forward movement of the worms.

**Figure 2:**
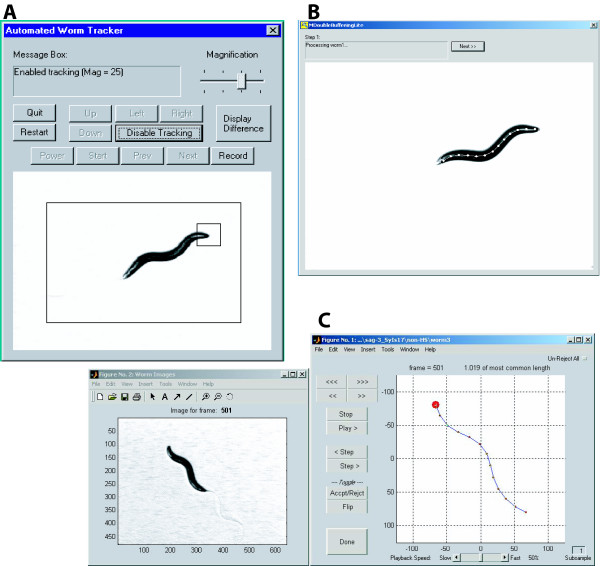
**User Interfaces**. A. Tracker User Interface. A simple GUI controls the tracking and recording. B. Recognizer2.1 User Interface. A simple user interface illustrates the progress of the recognition process. The image of the worm is shown with the spine and points superimposed. C. Wormproc User Interface. The interface for processing body posture shows a reference image of worm on plate to assist with worm orientation (left) and the main data processing control window (right) depicting an abstraction of worm during processing. If Recognizer2.1 inappropriately flips head and tail, it can be overridden with the Flip function. The program automatically rejects frames in which the worm length is outside of a calculated normal range, but these can be overridden with the Accpt/Rejct button, or all frames can be scored manually by hitting the Un-Reject All button.

With the videotaping apparatus powered and the Tracker program started on the desktop computer, a single young hermaphrodite is placed at the center of a prepared Petri dish without transferring excess food, and the dish is placed onto the carrier on the motorized microscope stage. The operator uses the joystick and/or Tracker on-screen controls to position the moving worm within a bounding box on the Tracker program's graphical user interface (GUI) and starts the tracking function of the program (Figure 2A).

Our Tracker program grabs images (frames) from the video stream from the camera looking for differences between successive frames that indicate a moving worm. Tracker identifies the changed regions as either newly occupied or newly vacated. If a changed region falls outside of a 240 × 160 pixel bounding box (within the 320 × 240 pixel image) Tracker sends a command to the motorized microscope stage controller to shift the stage half of a screen width and/or height to relocate the itinerant worm back to the middle of the camera's view field. With the computer re-positioning the worm as necessary, the video stream from the camera is recorded onto VHS tape for use in the data extraction step.

Tracker was designed to move the microscope stage in rapid, discrete shifts, allowing the worm to crawl to the edge of the view field before being reined back to the center of our camera's view field. We chose this protocol to eliminate the need for position feedback sensors and stage position data storage.

### Data extraction

Our Data Extraction apparatus consists of a personal computer with our Recognizer2.1 software package, a Matrox Meteor-II/Standard video frame grabber, a Panasonic model AG-5710P VHS video cassette recorder (which has an RS-232 connection for computer control and feedback of VCR operation), and an optional Sony video monitor (Figure 1B).

We use our Recognizer2.1 program to extract worm position and posture data from the video recording made in the previous step. Recognizer2.1 commands the VCR to play back segments of the worm videotape made previously and, using a double-buffering paradigm, grabs and processes images (frames) from the video stream at the rate of ~6 Hz. (Processing rate is a function of available computing bandwidth.) During extraction, Recognizer2.1 displays the grabbed/annotated images in its GUI window (Figure 2B).

Recognizer2.1 locates the worm in each 640 × 480 pixel image using contrast thresholding, identifies the worm's boundary curve, and uses the boundary curve to calculate the worm's "spine." The program mathematically distributes points along the length of the "spine" and records the X-Y position of the points in an output file. (*n *points define *n-1 *body segments, between which there are *n-2 *articulation points, or "bends", whose angles we calculate.) We typically set Recognizer2.1 to distribute 13 points (13 points: 12 body segments: 11 articulation points) along the worm's spine, but the program can be easily user-customized to apply as many or as few points as desired if, for example, a longer worm is analyzed.

The result of running Recognizer2.1 is a set of folders saved to hard disk, each containing a file called "points" containing the table of X-Y coordinates for the 13 points on the worm spine in each grabbed image, and a set of bitmap worm images. The X-Y coordinate table's rows are the data for each image, with the first pair of rows containing the coordinate data from the first image grabbed, and the last pair of rows containing the data from the last image. The table's columns are the data for each of the points distributed along the worm's spine, but since Recognizer2.1 does not identify head versus tail, the saved coordinate data simply represents the posture and screen position of the worm in each processed image without regard to head-tail orientation. Data "orientation" is performed as part of the data processing phase.

We assume the time between successive grabbed and processed images is consistent within a data set, and calculate the effective grab rate as the number of grabbed frames in the data set divided by the length of the data set (in seconds). Measurements of the distribution of inter-grab intervals were made by saving computer-generated timestamps corresponding to the X-Y coordinate data normally saved. The distribution of intervals indicates that there is less than 10% variability and thus this only accounts for a small fraction of the observed variability within observations of each animal (see Figure 4 below). Videotape stretch before or during playback could also be a source of variability but we assumed this to be negligible.

**Figure 3:**
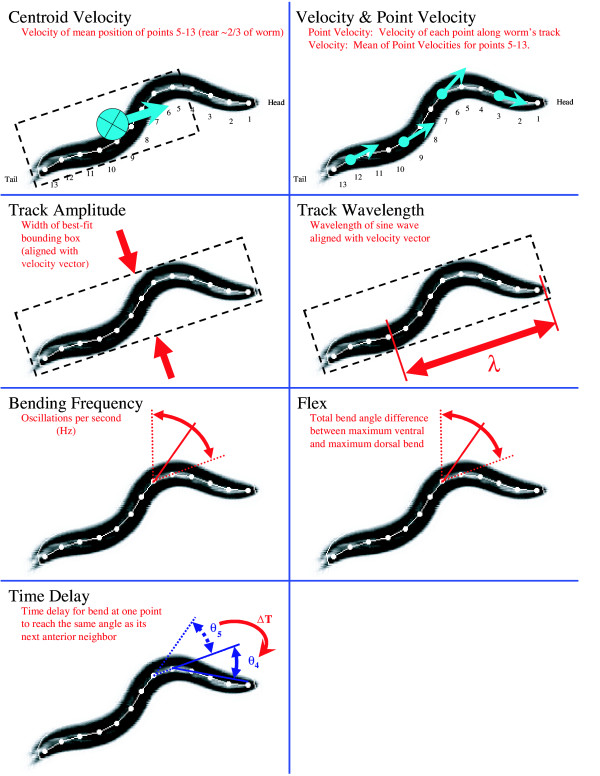
**Sample Attributes**. The key attributes that are extracted by Wormproc program are shown schematically. Centroid velocity is the translation of the mean position of the rear two-thirds of the animal. Point velocity is the velocity of each point along the animal's track; velocity is the mean of the point velocities for points 5–13. Track amplitude is the maximum width of a box around the worm. Track wavelength is the length of the sine wave that fits the worm's posture. Bending frequency is the frequency of oscillations between adjacent segments. Flex is the maximum difference in angle between the ventral- and dorsal-most flexion at each articulation point. Time delay is the time required to propagate flexion between adjacent articulation points.

**Figure 4:**
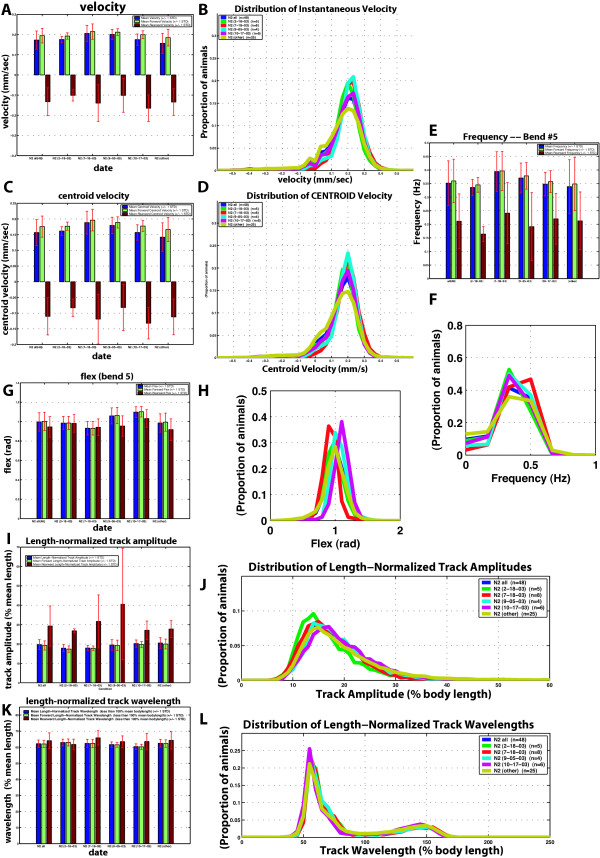
**Variability of wild-type movement**. For each metric, the aggregate statistics are shown along with individual days' experiments. Each daily group is designated by date. The 'other' group comprises 25 individuals that were tested on 16 different days in groups of 1–3. For each metric, a bar graph of the means is displayed (A, C, E, G, I, K) as well as histograms (B, D, F, H, J, L). For bar charts: Blue, mean; green, forward; red, backwards. For histograms: Blue, all 48 N2 animals (n = 48); Green, 2-18-03 dataset (n = 5); Red, 7-18-03 dataset (n = 8); light blue, 9-05-03 dataset (n = 4); magenta, 10-17-03 dataset (n = 6); yellow, other dataset (n = 25). n = number of individuals tested; n is the same for all panels. A. Mean velocity. B. Velocity histogram. C. Mean centroid velocity. D. Centroid velocity histogram. E. Mean frequency at bend 5. F. Frequency at bend 5 histogram. G. Mean flex at bend 5. H. Flex at bend 5 histogram. I. Mean length-normalized track amplitude. J. Length- normalized track amplitude histogram. K. Mean length-normalized wavelength. L. Length-normalized wavelength histogram. Each histogram curve represents the distribution of 632 to 1485 individual measurements per worm.

### Data processing and analysis

Data processing and analysis is performed using a suite of programs we developed in Matlab (from The MathWorks) comprised of three applications: "*Wormproc*" (worm processing), "*Metrics*", and "*Histograms*." Data processing proceeds in several steps.

### Wormproc

The researcher runs the "Wormproc" program for each worm to convert the X-Y data into a usable format. First, Wormproc loads into RAM the X-Y coordinate data and images captured and saved to disk by Recognizer2.1. Since Recognizer2.1 does not distinguish between head and tail, Wormproc orients spines by selecting the spine orientations (either oriented "as recorded" or "reversed") that are minimally different from each preceding spine. Further, Wormproc flags spines with lengths that are outside of a calculated "normal" range (asserting that they are invalid or missing data), and identifies the end of the worm that moves the most as the head-end (based on typical *C. elegans *foraging behavior). Disjointed data segments, for example before and after an omega bend, are treated as separate data segments; Wormproc identifies head position before and after such breaks.

Next, Wormproc mathematically removes the microscope stage shifts from the X-Y data; the program recognizes stage shifts by a velocity spike (a very large displacement between two consecutive frames) uncharacteristic of a nematode. The program offsets the X-Y coordinate data after each stage shift to continue the worm's path of locomotion, interpolating over any single missing frames. Occasionally Recognizer2.1 will have grabbed a worm image while the microscope stage is moving. In these instances Recognizer2.1 either will not be able to identify any worm in the image or, because of the interlaced video and contrast, will only be able to recognize a tiny area of the smeared image as worm. In either case the X-Y data for these frames will have been automatically rejected for being outside of the normal length range for the worm.

Finally, Wormproc provides a GUI (Figure 2C) that allows the user to verify (and modify, if necessary) the computer's assertions on valid/invalid data, and worm head/tail orientations via an animation of the subject worm's movements with still images presented in a second window for reference. When the user is satisfied, the program saves the oriented and verified data to hard disk.

### Metrics

We developed a software application called Metrics which extracts useful measures of nematode locomotion and morphology from the processed X-Y coordinates for each worm. For each worm we extract eleven attributes (Figure 3).

One set of attributes concerns the speed of worm movement. We calculate the instantaneous speed of the animal's centroid (its 'centroid velocity' or VELC). We define the centroid as the mean position of points 5–13 (approximately the posterior two-thirds of the body), and the instantaneous centroid velocity as the change in centroid position over time. Likewise, we calculate the instantaneous velocities of all 13 points along the spine as they move over time (the point velocity, or PTVEL). We define the means of the point velocities for points 5–13 as the worm's 'velocity' (VEL). Instantaneous velocity, point velocity and centroid velocity are identified as 'forward' (positive) or 'backward' (negative) reflecting the direction the animal is moving. The MODE lists the instantaneous movement direction with 1's (forward) or -1's (backward). MODE is determined automatically in Metrics by evaluating whether the majority of points 5–12 are moving closer to their anterior or to their posterior neighbors through successive frames. Due to signal noise, MODE cannot at present be set to "no movement". THETA is the instantaneous velocity (VEL) vector direction.

A second set of attributes concerns propagation of the contractile wave. The flex (FLEX) is the difference between maximum positive and negative bend angles in a sliding time window for each of the articulation points. The bending frequencies (FRE) are the time-windowed bending frequencies at each of the worm's articulation points. Time delay (PHS) is a matrix containing the time delay required for an articulation point to reach the same angle as its next anterior neighbor; this metric describes the rate of wave propagation along the worm.

A third set of attributes describes the worm's waveform. The track amplitude (AMPT) is the instantaneous worm track waveform amplitude, specifically the width of a best-fit bounding box aligned with the worm's instantaneous velocity vector. Wavelength (WAVELNTH) is a measure of the instantaneous physical wavelength of the worm's sinusoidal body posture.

Another attribute describes morphology. The worm's length (LEN) is the sum of the distances between the points along the worm's "spine."

### Histograms and other data visualization routines

We developed several data visualization routines to display and compare the movement attributes of worms.

The most common program we use is "Histograms" which displays a set of histograms for comparing locomotory parameters for populations of nematodes. The standard attributes displayed are: Centroid Velocity, (Mean Point) Velocity (velocity of the worm along its sinusoidal track), Flex (for several articulation points), Bending Frequency (for several articulation points), Time Delay (for several articulation points), Track Wavelength, and Track Amplitude (both in millimeters and normalized as a percent of mean worm body length).

In addition to comparing populations of worms, it is often useful to compare individual worms within a population, for which we developed "iHistograms." This application produces the same charts as "Histograms," but displays the data for individual worms instead of populations.

Using the flexibility of the Matlab programming environment we have developed a multitude of specialty analysis tools, ranging from toxicant concentration-response curves, to speed decay as a function of time, to animation routines to visualize wave propagation, to reversal frequency. With a bit of creativity, output can be customized to a broad range of experiments. To demonstrate the general applicability of this type of system to nematode biology, we provide a few salient examples: genetics and toxicology.

### Reproducibility of data

One common problem in behavioral studies on *C. elegans *is day-to-day variability. To test whether data obtained on different days could be pooled, we analyzed the movement of small numbers of wild-type individuals on different days. We then compared the means for each movement parameter of each daily group to those for the pooled total. Specifically, we compared seven groups comprising four-twelve individuals to the total data set of 58 individuals. Two groups, with five animals each, had means for more than one parameter that were significantly different from the pooled total and were eliminated from our analysis. For the remaining groups, the p values for all parameters except FLEX ranged from 0.07 to 0.99. For two of the included groups, mean values for FLEX for the more posterior articulation points were significantly different from the pooled total. We therefore report FLEX measurements for a more anterior articulation point (bend 5) only. Comparison of the daily groups to the pooled total is displayed in Figure 4, which shows types of graphical representation available in our system.

### Genetic analysis

**Figure 5:**
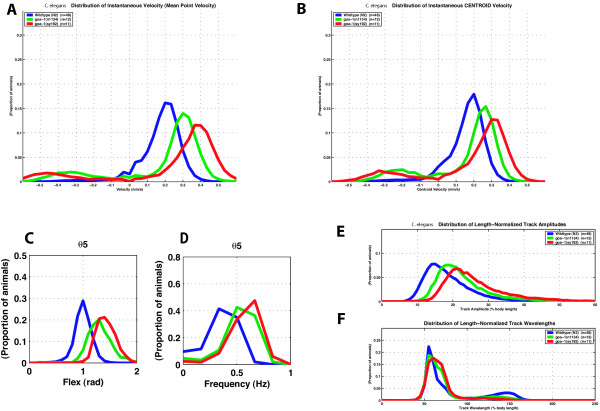
**Comparison of two alleles of *goa-1***. Blue, wild-type (n = 48); green, *goa-1 (n1134)*, a null allele (n = 12); red, *goa-1 (sy192)*, an antimorphic allele (n = 11). n = number of individuals tested; n is the same for all panels. A. Distribution of velocity. B. Distribution of centroid velocity. C. Flex at bend 5. D. Frequency at bend 5. E. Length-normalized track amplitude. F. Length-normalized track wavelength. Each curve represents the distribution of 632 to 1485 individual measurements per worm. The population mean values reported in the text reflect only forward moving worms and are based on 550 to 1454 individual measurements per worm.

We tested whether the system was useful for comparing alleles of the same gene, and alleles of different genes that result in qualitatively similar movement phenotypes. *goa-1 *encodes the only Go-alpha subunit in *C. elegans *and is involved in locomotion [18,26,27,46]. *goa-1(n1134) *is a reduction-of-function allele, defective in the consensus sequence for myristoylation at the amino-terminus [27], and is protein-negative on a Western blot [47]. *goa-1(sy192) *is an antimorphic allele [48]. Although *n1134 *and *sy192 *homozygous mutant animals look very similar by visual examination, quantification of their movement revealed that s*y192 *affects certain movement parameters more severely than *n1134 *(Figure 5). The mean forward point velocity of *sy192 *is 0.37 (+/- .04) mm/sec versus 0.29 (+/- .06) mm/sec for *n1134 *and 0.20 (+/- .04) mm/sec for wild type (Figure 5). These values are significantly different (p < 0.001 for each pair). The centroid velocities show the same relationship *sy192 *>*n1134 *> wild type. The flex is also significantly different: *sy192 *has a flex at articulation point (bend) 5 of 1.4 (+/- .05) radians, *n1134 *1.3 (+/- .09) radians and N2 1.0 (+/- .09) radians (p < 0.0005 for all pairs). The frequencies of *n1134 *and *sy192 *hermaphrodites, however, are similar: 0.58 (+/- .06) Hz at bend 5 for *sy192 *versus 0.53 (+/- .10) Hz for *n1134 *(p = 1.08) versus 0.36 (+/- .08) Hz for wild type (p < 0.0005). The track amplitudes of all three genotypes are significantly different. When normalized for body length, *sy192 *has a track amplitude of 25.53 (+/- 1.14) % body length, *n1134 *22.00 (+/- 1.64) % body length, and N2 19.27 (+/- 2.34) % body length (p < 0.0005 for all pairs). The wavelengths, however, are not different for all genotypes. *sy192 *has a wavelength significantly different from wild type (64.19 +/- 1.40 % body length versus 62.03 +/- 2.28 % body length, p = 0.004). However, the wavelength of *n1134 *(63.39 +/- 2.3 % body length) is not different from N2 (p = 0.07) or from *sy192 *(p = 0.34). In summary, mutations in *goa-1 *that cause hyperactive movement increase both the point and centroid velocities, increase the flex of articulation points, increase the frequency of body bends, and increase the track amplitude compared to wild type. With the exception of frequency, the antimorph *sy192 *has a more profound effect on these parameters than the null mutation *n1134*. *sy192 *also decreases the wavelength compared to wild type, but *n1134 *has only a mild effect on wavelength.

**Figure 6:**
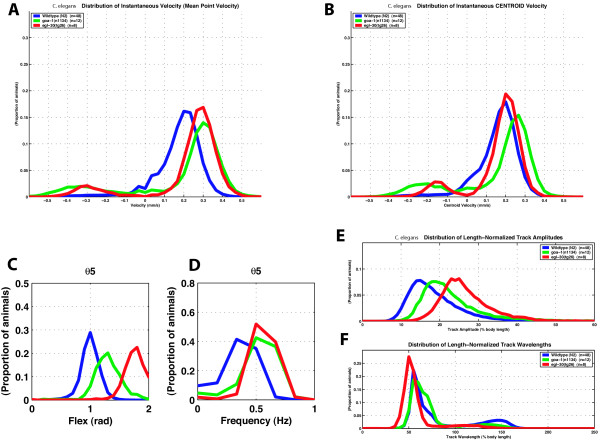
**Comparison of *goa-1 *loss-of-function and *egl-30 *gain-of-function mutations**. Blue, wild-type (n = 48); green, *goa-1 (n1134)*, a null allele (n = 12); red, *egl-30 (tg26)*, a gain-of-function allele of *egl-30 *Gq (n = 8). n = number of individuals tested; n is the same for all panels. A. Distribution of point velocity. B. Distribution of centroid velocity. C. Flex at bend 5. D. Frequency at bend 5. E. Length-normalized track amplitude. F. Length-normalized track wavelength. Each curve represents the distribution of 632 to 1485 individual measurements per worm. The population mean values reported in the text reflect only forward moving worms and are based on 550 to 1454 individual measurements per worm.

For movement, *goa-1 *acts antagonistically to *egl-30*, thus increased *egl-30 *activity is similar to loss of *goa-1 *activity [28,29]. We therefore compared the movement of worms bearing a strong *egl-30 *gain-of-function allele, *tg26 *[49,50], to those lacking *goa-1 *activity (*goa-1(n1134)*). Visually, *tg26 *mutants move with more exaggerated body bends than both wild type and *n1134*. This difference was detected by our movement analysis system (Figure 6). The mean forward point velocity of both *n1134 *and *tg26 *are similar (0.28 +/- .03 mm/sec for *tg26 *and 0.29 +/- .06 mm/sec for *n1134*, p = 0.76), and faster than wild type (0.20 +/- .04 mm/sec, p < 0.0005). Although not statistically significant, the forward centroid velocity for *tg26 *(0.21 +/- .02 mm/sec) is more similar to that of N2 (0.18 +/- .03 mm/sec, p = 0.02) and less similar to that of *n1134 *(0.24 +/- .05 mm/sec, p = 0.05) than the mean forward point velocities. This difference reflects the increased path length any point on the spine of the *tg26 *mutant must travel to displace the centroid. Both mutations cause increased flex and frequency compared to wild type. The flex of *tg26 *at bend 5 was 1.77 (+/- .04) radians versus 1.27 (+/- .09) radians for *n1134 *(p < 0.0005), and 1.00 (+/- .09) radians for wild type (p < 0.0005). The frequency of *tg26 *at bend 5 was 0.56 (+/- .05) Hz versus 0.53 (+/- .10) Hz for *n1134 *(p = 0.37), and 0.36 (+/- .08) Hz for wild type (p < 0.0005). While both alleles affect frequency similarly, *tg26 *has a more profound effect on flex than does *n1134*. *tg26 *also has a more profound effect on amplitude than does *n1134*. When normalized for body length, *tg26 *has an amplitude of 26.08 (+/- .67) % body length compared to 22.00 (+/- 1.63) % body length for *n1134 *(p < 0.0005) and 19.27 (+/- 2.36) % body length for wild type (p < 0.0005). The track wavelength for *tg26 *is shorter than that of *n1134 *and N2: 54.04 (+/- 1.13) % body length for *tg26*, 63.4 (+/- 2.33) % body length for *n1134 *(p < 0.0005), and 62.3 (+/- 2.28) % body length for wild type N2 (p < 0.0005).

Our movement analysis system is thus able to discriminate between the effects of different mutations that affect the same parameters of movement.

### Toxicology

To test the utility of our system for analyzing the effects of toxicants, we focused on the neurotoxin aldicarb and the metabolic inhibitor arsenite. We first established baseline conditions for these toxins. We then tested whether existing mutations would increase the sensitivity to these compounds. We focused on mutations that affect cuticle and P-glycoprotein transporters.

**Figure 7:**
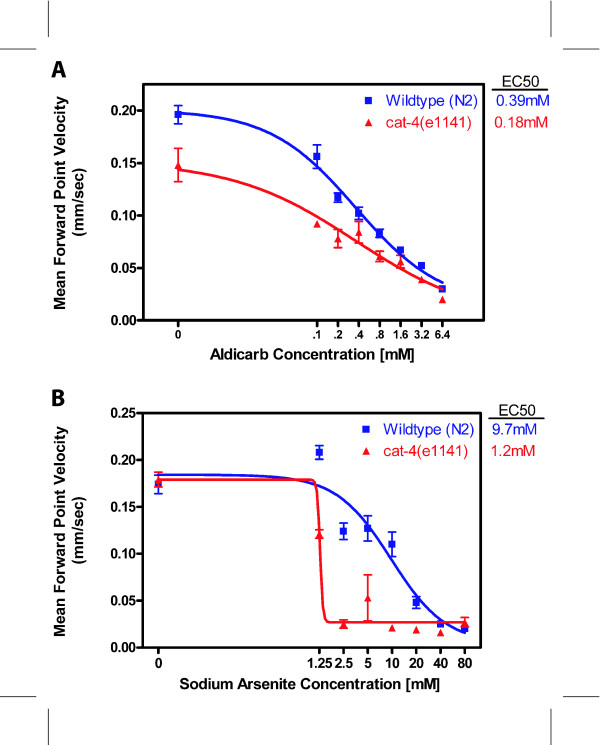
**Toxicant sensitivity of wild-type and *cat-4***. A. Sensitivity to aldicarb. For N2, n = 16 animals for 0 mM aldicarb and 17 for all other concentrations. For *cat-4*, n = 6 for all concentrations. B. Sensitivity to arsenite. For N2, n = 17 animals for 2.5 mM sodium arsenite and 18 for all other concentrations. For *cat-4*, n = 4 for all concentrations.

We analyzed *C. elegans *movement in the presence of increasing concentrations of aldicarb (2-methyl-2-(methylthio)propionaldehyde O-methylcarbamoyloxime) and sodium-arsenite (NaAsO_2_). We first determined that a 30-minute exposure to 6.4 mM aldicarb induced near paralysis in wild-type *C. elegans*. We then recorded movement of individual hermaphrodites following a 30-minute exposure to aldicarb concentrations from 0 to 6.4 mM. We tested 16–17 individual wild-type worms for each concentration of aldicarb and found that the concentration reducing wild-type mean forward point velocity by 50% (EC50) is 0.39 mM aldicarb (Figure 7A). We similarly determined that a 3-hour exposure to 80 mM sodium-arsenite induced paralysis in wild-type worms. We have determined an EC50 of 9.7 mM NaAsO_2 _for wild-type *C. elegans *(Figure 7B). Of the movement parameters tested, mean point velocity, centroid velocity, track amplitude and track wavelength were equally sensitive to aldicarb, with differences apparent at the lowest concentration tested (0.1 mM). A reduction of frequency was seen at 0.2 mM, but flex was resistant to the effects of aldicarb and alterations were only apparent at the highest concentrations (data not shown). For sodium arsenite, most parameters were affected after exposure to 2.5 mM, except for flex which was only affected at concentrations above 20 mM (data not shown).

We tested three candidate hypersensitive mutant *C. elegans *strains for movement in response to increasing doses of aldicarb and sodium-arsenite.

The *cat-4 *gene encodes GTP cyclohydrolase I (C. Loer, personal communication; see also [51]) necessary for biosynthesis of biogenic amines; it is hypersensitive to several disparate agents such as the neurotransmitter serotonin and the detergent SDS, suggesting a weaker or more porous cuticle (C. Loer, pers. comm.). *cat-4 *mutants are 2.2-fold more sensitive to aldicarb (EC50 = 0.18 mM vs. 0.39 mM; Figure 7A) and 8.1 fold -fold more sensitive to arsenite (EC50 = 1.2 mM vs. 9.7 mM; Figure 7B) than wild-type *C. elegans*. Only the EC50s of wild type and cat-4 on arsenite are significantly different.

**Figure 8:**
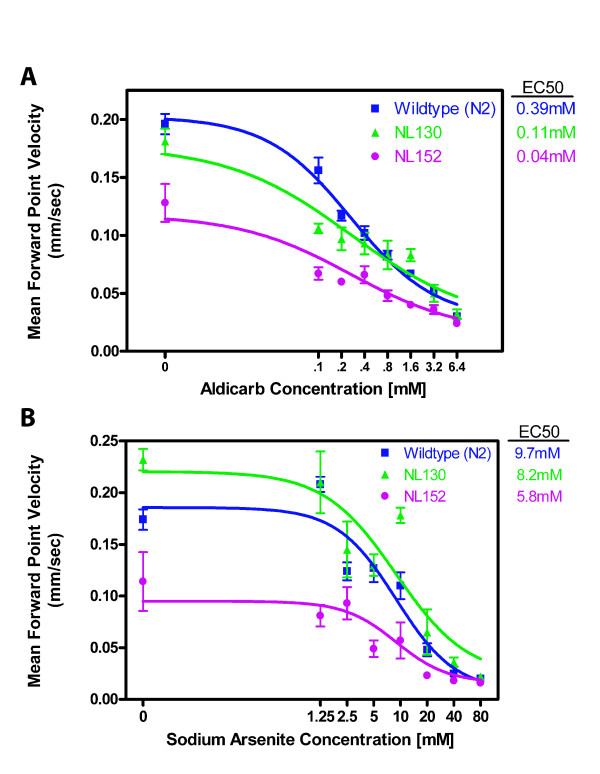
**Toxicant sensitivity of NL130 and NL152**. A. Sensitivity to aldicarb. For N2, n = 16 animals for 0 mM aldicarb and 17 for all other concentrations. For NL130 and NL152, n = 6 for all concentrations. B. Sensitivity to arsenite. For N2, n = 17 animals for 2.5 mM sodium arsenite and 18 for all other concentrations. For NL130 and NL152, n = 4 for all concentrations.

P-glycoproteins are membrane transporters in certain cells that protect the cell against environmental toxins by moving such agents out of the cell. The strain NL130 carries deletions for two P-glycoproteins encoded by *pgp-1 *and *pgp-2 *[52]. Mutants lacking these two P-glycoproteins are hypersensitive to colchicine and chloroquinone. We have shown that NL130 is 3.5-fold more sensitive to aldicarb (EC50 = 0.11 mM vs. 0.39 mM; Figure 8A) and 1.2-fold more sensitive to arsenite (EC50 = 8.2 mM vs. 9.7 mM; Figure 8B) than wild-type *C. elegans*. The strain NL152 carries deletions for *mrp-1 *as well as those for *pgp-1 *and *pgp-3 *[53]. The *mrp-1 *gene encodes a *C. elegans *homolog of the mammalian multidrug resistance-associated protein (MRP), another transporter that protects cells from toxins. NL152 also showed increased sensitivity (9.7-fold) to aldicarb (EC50 = 0.04 mM vs. 0.39 mM; Figure 8A), and only slightly increased sensitivity to arsenite (EC50 = 5.8 mM vs. 9.7 mM; Figure 8B). However, NL152 is also somewhat movement impaired in the absence of toxic agents.

Our system is thus useful for analyzing the effects of toxicants on nematode movement and for examining the effects of genetic background on toxicant sensitivity.

## Discussion

We describe a usable automated system to record and analyze *C. elegans *locomotion and to display quantitative data. We describe several useful parameters of locomotory behavior. These include automatic determination of the velocity of the worm's centroid, the velocity along its track, the degree of flex of body at various positions along the body axis, the bending frequency between adjacent segments, and the body-length normalized track amplitude and wavelength (Figure 3). Many of these measurements match what *C. elegans *geneticists have typically observed in describing movement variations such as velocity and body bends per minute. Our system thus provides facile quantification of standard phenotypes. Additionally, our system provides measurements that describe many aspects of movement that relate to underlying neural and mechanical mechanisms of locomotion such as propagation of the contraction wave. We envision that the ability to describe in a quantitative and automatic manner nematode movement will facilitate modeling of *C. elegans *movement.

We have demonstrated that our system can be used to distinguish between different alleles of the same gene, different genes, and different environmental conditions. It thus provides a rich dataset for analysis of gene function and toxicology. In addition, this system can be adapted to score other phenotypes. For example, the frequency of spontaneous reversals can be easily extracted from the data set. This system gives worm length as well, and thus we can normalize the track wavelength. Measurement of body length has been used to screen for suppressor mutations and to analyze mutations that affect gene expression [47,54], and thus this system can be used for genetic studies besides behavior.

The basic platform described here might be readily extensible to analysis of other nematode behaviors such as male mating, as well as the behavior of other organisms such as the crawling of insect larvae. Although the system is not high throughput, recording of each individual hermaphrodite takes only seven minutes of largely hands-off time on the part of the researcher. Including processing of the data, a single data set comprising 10–12 individuals can be generated in less than three hours.

There are a number of limitations to our system. We use all relative coordinates, and thus tracking worms with respect to specific locations on the Petri plate, for example a gradient of chemoattractant, is not possible. In addition, the noise inherent in our tracking system, most likely arising from its use of analog video, precludes our detecting a worm that is definitively not moving. Moreover, the throughput of our system is limited by its inability to follow multiple worms simultaneously.

While preparing this manuscript, we compared our system to that of W. Schafer and colleagues [43,44], and identified useful features of each system. We have begun to develop a joint system taking advantage of the best features of each system to allow further software development to proceed in an efficient manner. A prototype has been described [45], but the system described here has been used in a number of ongoing studies in our laboratory and is still in continual use.

## Methods

### Strains and media

*C. elegans *N2 [1]. NL131 *pgp-3(pk18)X *[52]; *goa-1(sy192)I *[26]; *goa-1(n1134)I *[27]. CB1141 *cat-4(e1141*)*V*[55]. NL130 *pgp-1(pk17)IV; pgp-3(pk18)X *[52]. NL152 *pgp-1(pk17) IV; pgp-3(pk18) X; mrp-1(pk89)X *[53]. Nematode Growth Medium (NGM) is from Brenner [1].

### Preparation of plates for observation

10 cm NGM recording plates are equilibrated to 20°C for 18–20 hours prior to being spread with bacteria. Approximately one hour before beginning our recordings, 600 μl of fresh OP50 overnight culture is spread onto each plate, rapidly swirling and shaking to achieve a thin, featureless lawn of food across the entire surface. Excess solution is drawn from the edge with a Pipetman. Each food-spread plate is covered with a tissue (Kimwipe), to ensure that the plate remains dust-free as it dries. The food is allowed to dry onto the NGM surface just until the surface exhibits a matte finish (about 45 minutes), at which time the tissues are replaced by the Petri dish lids and the plates are ready for use. The time required for drying is monitored as a crude measure of the relative moisture content of the plates. Plates requiring more than 60 minutes to dry are discarded. Each plate is used within three hours of drying.

### Assay conditions

L4 hermaphrodites are selected 18–20 hours prior to recording to control for age. Individuals are placed on assay plates and the plate is placed in the holder on the microscope stage. After two minutes the worm is located and recording begins. Each worm is recorded for five minutes and the central four minutes of data are analyzed. Incubations and recordings are done in a constant temperature room at 20°C.

### Toxicant treatment

Aldicarb and sodium-arsenite stocks were prepared in H_2_O (at 55°C for aldicarb), and the appropriate volume added to cooled NGM media prior to pouring plates. The volume of added solution was kept constant. The pH of media containing sodium arsenite was adjusted to 5.8–6.0 (to match NGM without toxicant) with concentrated HCl. This was not necessary for plates containing aldicarb. 10 cm assay plates and 5 cm pre-incubation plates were prepared similarly and stored at 4°C until needed. To insure an ample source of food during pre-incubation, 5 cm plates were seeded with fresh OP50 18–20 hours before use and stored at 37°C until 2 hours prior to the assay when they were placed at 20°C. 10 cm assay plates were equilibrated to 20°C and spread with a thin lawn of OP50 as described above. Hermaphrodites were placed on pre-incubation plates and incubated for 30 minutes for aldicarb and 3 hours for sodium-arsenite prior to recording. Following pre-incubation, individuals were transferred to assay plates and recorded after a 2-minute rest on the microscope stage as described above. All preincubations and assays were performed at 20°C.

### Microscope

Our recordings were made using a Wild M5A stereo dissecting microscope with a 25× objective lens and a 1.25× camera mount.

### Hardware and software

Tracker and Recognizer2.1 are written in Microsoft Visual C++ (6.0), using Matrox ActiveMIL-Lite libraries for image manipulation from the Matrox Meteor-II frame grabbers.

Tracker is programmed to work with the optics present on our Wild microscope but can be customized to work with other microscopes with minor software changes reflecting the appropriate magnification levels. For our microscope, each stage shift commanded by Tracker to re-center a worm is between 0.362 mm and 0.904 mm depending on the orientation and speed of the worm. Our BioPoint controller is programmed to move the stage with a starting speed of 6.3 mm/sec (= 10,000 pulses/sec with a 0.628 μm/pulse step size), an acceleration rate of 44.8 mm/sec^2 ^(71,400 pulses/sec^2^), and a maximum run speed of 31.4 mm/sec (50,000 pulses/sec). However, because of the small distances traveled, we expect the stage to reach a maximum velocity of 8.5 mm/sec (13,500 pulses/sec) during a stage shift.

Recognizer2.1 calculates the location of the user-defined number of points (typically 13) along each worm's spine as follows: Recognizer2.1 identifies the center of the darkest portion of the image as its focus of attention and extracts a portion of the image surrounding the center point for further processing. Next, the extracted gray-scale image is turned into a binary image using a segmentation algorithm. The segmentation routine compares the ratio of pixel values resulting after applying two smoothing filters (sum of squared pixel values and square of summed pixel values, both in a typically 15 × 15 pixel neighborhood) against a user defined threshold. Pixels with pixel value ratios less than the threshold are "worm" while the others are "background." Connected regions in the binary image are labeled and Recognizer2.1 then selects the largest-area connected region as the worm in the image. Recognizer2.1 calculates the worm's boundary polygon with a user-defined number of vertices – typically 50 – by interpolating equidistant vertices along the chain of pixels on the perimeter of the worm region. Boundary polygon vertices are passed to Triangle [56], which generates constrained Delaunay triangulations across the worm boundary polygon. Finally, Recognizer2.1 connects the circumcenters of the resulting triangles to form segments of the worm's spine curve, along which Recognizer2.1 interpolates the 13 equally spaced "spine" points.

Feature extraction tools are written in Matlab and C++. Attributes are calculated as follows, described based on a typical analysis distributing 13 points along a worm's spine:

#### Speed attributes

##### Centroid Velocity (VELC)

Centroid velocity is a series of speed values, one for each pair of successive frames, and is calculated as the distance the worm's centroid moves between successive frames divided by the time between successive frames. We define the worm's centroid as the mean position of points 5–13 (approximately the rear two-thirds of the worm).

VELC = (Δ Centroid Position) / (Δ Time)

Sign of VELC describes forward (positive) or backward (negative) movement. Movement of the anterior one-third of the worm is ignored from this calculation (and the VEL calculation below) to minimize the effect of foraging behavior on speed.

##### Point Velocity (PTVEL)

Point velocity is a matrix of speed values, one for each of the 13 points for each pair of successive frames, calculated as the distance each point along a worm's spine moves between successive frames divided by the time between successive frames.

PTVEL = (Δ Point Positions [all 13 points]) / (Δ Time)

Signs of PTVEL describe each point's forward (positive) or backward (negative) movement. PTVEL describes the speed with which each point travels along the worm's serpentine path.

##### Velocity (VEL)

Velocity is a series of speed values, one for each pair of successive frames, calculated as the distance each point along a worm's spine moves between successive frames divided by the time between successive frames.

VEL = Mean of PTVEL's 5–13 for each pair of successive frames

Sign of VEL describes forward (positive) or backward (negative) movement. VEL describes mean speed of the worm's body along its sinusoidal path.

##### Forward or Backward Direction (MODE)

MODE is a series of flags indicating the signs of a worm's instantaneous speed attributes (VEL and VELC), that is, whether the worm is moving forward or backward. Mode is calculated automatically at the same time as speed attributes by comparing:

D1: mean of the distances between points 4–11 at time τ and their posterior neighbors, points 5–12 at time τ + 1

with

D2: mean of the distances between points 6–13 at time τ and their anterior neighbors, points 5–12 at time τ + 1

Forward movement is indicated by D1<D2; backward by D2<D1. The MODE flags can be either 1 (forward) or -1 (backward). (Note that the points distributed along a worm's spine are equidistant, so a non-moving worm would have no difference in the distance between neighboring points over time.)

##### Instantaneous Velocity Vector Direction (THETA)

The instantaneous velocity vector direction is calculated as the direction the worm's centroid moves between successive grabbed frames. (Calculated as:

THETA = arc tangent of the worm's X-Y displacement.)

#### Wave propagation attributes

##### Flex (FLEX)

We calculate the matrix of angles between each segment (that is, at each articulation point) for each frame by:

angle = acos [(V1x*V2x + V1y*V2y) / (|V1|*|V2|)]

where

V1 = first segment vector and

V2 = second segment vector

We define the FLEX at each articulation point as the maximum angle *difference *during each possible 32 frame (~6 second) time window; that is, the most positive angle minus the most negative angle.

##### Bending Frequency (FRE)

We apply the spectrogram function ("specgram") from Matlab's Signal Processing Toolbox to the matrix of bend angles calculated for FLEX. Specgram calculates a windowed discrete-time Fourier transform (short-time Fourier transform) for the changing angles for each articulation point using a sliding window 32 frames (~6 seconds) wide. We quote from Matlab's documentation for specgram: "*specgram calculates the spectrogram for a given signal as follows: 1. It splits the signal into overlapping sections and applies the window specified by the window parameter to each section. 2. It computes the discrete-time Fourier transform of each section with a length nfft FFT to produce an estimate of the short-term frequency content of the signal; these transforms make up the columns of B. The quantity (length(window) – numoverlap) specifies by how many samples specgram shifts the window. 3. For real input, specgram truncates the spectrogram to the first nfft/2 + 1 points for nfft even and (nfft + 1)/2 for nfft odd*." The magnitude of the function indicates the relative energies of the signal's component frequencies. We take the highest magnitude (non-constant) component frequency as the characteristic frequency of that time-window of angles. (If two or more frequencies share the highest magnitude, the lower frequency is identified as the characteristic frequency.)

##### Time Delay (PHS)

For the time delay calculation, the program correlates anterior bend angles with posterior bend angles occurring at later time using a Dynamic Time Warping function. The program then uses these correlations to calculate the time required for the posterior bend to reach the same angle as its anterior neighbor.

#### Track waveform attributes

##### Track Amplitude (AMPT)

The program aligns the major axis of a best-fit bounding box with the worm's instantaneous velocity vector. The width of the bounding box (its minor axis) is taken as the instantaneous wormtrack waveform amplitude.

##### Track Wavelength (WAVELNTH)

We apply a rotation and translation transform to the spine of the worm from every frame to mathematically align each worm with y = 0 using the instantaneous velocity vector as the worm's centerline:

We create a matrix w containing the XY coordinates of the 13 points for a worm's spine and create a translation transform that we will use to center the worm's midpoint at the XY origin:



We also create a rotation transform to align the instantaneous velocity vector (with angle theta), to y = 0:



We multiply the two matrices to create a convenient combined transform

C = B*A;

and finally multiply our matrix by the combined transform matrix

ww = C*w;

which yields a matrix ww with the rotated and aligned coordinates for the 13 points. We perform a spatial Fast Fourier Transform (FFT, using Matlab's built-in fft function) on the rotated "spine" of each worm, using the varying y-values as the "signal" with their corresponding x-position values defining the signal to be in a spatial (rather than temporal) domain. By working in the spatial domain, the result from our FFT is in cycles per mm. The inverse of this result is the track wavelength, in mm per cycle.

#### Morphology attribute

##### LEN (spine length)

Sum of the distances between the points distributed along the worm's spine with each point-to-point distance calculated by (Δ x^2 ^+ Δ y^2^)^0.5^

Data Analysis and Comparison tools are written in Matlab. Our most common tool is called Histograms, which produces a set of charts displaying distributions of measures of behavior: Velocity, Centroid Velocity, Bending Frequency, Flex, Time Delay, Track Amplitude, Track Wavelength, Length-Normalized Track Amplitude, and Length-Normalized Track Wavelength.

To create a histogram curve for a given metric, for example a velocity histogram curve, we sort (or 'bin') each worm's velocity data into discrete ranges (or 'bins'). For velocity we use 'bins' that are 0.03333 mm/sec wide, i.e. bins would represent 0 to 0.03333 mm/sec, another 0.03333 to 0.06666 mm/sec, and so on. Bin sizes for each parameter are as follows: Point and Centroid Velocity, 0.03333 mm/sec; Flex, 0.1 radian; Frequency, 0.16665 Hz; Time Delay, 0.075 sec; Track Amplitude, 0.01 mm; Track Wavelength, 0.05 mm; Length-Normalized Track Amplitude, 1% mean body length; Length-Normalized Track Wavelength, 5% mean body length.

Again using our velocity example, the frequency of occurrence for the number of velocity values in each bin is normalized to a percent of velocities observed for that worm, and the normalized velocity distribution is added to a list of the other normalized velocity distributions for that population. (Normalizing each worm's data affords each worm equal mathematical significance.) The final histogram curve for the population is generated by plotting the mean value of each data bin's normalized frequency of occurrence (on the y-axis) versus the bin value (on the x-axis) which shows us the proportion of worms that exhibited each velocity. The same method is used for creating the histogram curves for each metric, naturally selecting bin sizes appropriate for the data in question:

### Statistical analysis

Standard statistical tests were performed using Matlab functions. Each p-value reported was from a one-way analysis of variance (ANOVA) comparing the distribution of mean values (one per individual) from each population of worms against that of each other population; each ANOVA tested the null hypothesis that the mean of the mean values from each populations were the same. Unless otherwise noted, statistical tests were performed on data from worms only when moving forward. For the toxicant concentration-response data, curves were fit by non-linear regression using Prism (GraphPad Software) sigmoidal dose-response equation with variable slope.

## Availability of source code

Source code is available through a GPL at 

Documentation of this software is available as a pdf from 

## Contributions of authors

SM, JB, JM and PS conceived and designed the original automated system for tracking and movement measurement. SM developed a working prototype system and brought in the key algorithms used. CC wrote most of the code in the current release. JM developed the protocol for the plate assay, and devised the dose-response and genetic experiments. RS conceived of applying automated analysis of *C. elegans *movement for toxicology studies, YK performed most of the toxicology experiments. JM, CC, RS and PS analyzed the data. CC, JM, and PS wrote the paper. All authors read and approved the final manuscript.
